# Proton-Enhanced Dielectric Properties of Polyoxometalates in Water under Radio-Frequency Electromagnetic Waves

**DOI:** 10.3390/ma11071202

**Published:** 2018-07-13

**Authors:** Shuntaro Tsubaki, Shogo Hayakawa, Tadaharu Ueda, Tomohiko Mitani, Ei-ichi Suzuki, Satoshi Fujii, Yuji Wada

**Affiliations:** 1 School of Materials and Chemical Technology Tokyo Institute of Technology, Ookayama 2-12-1 E4-3, Meguro, Tokyo 152-8550, Japan; tetuya.utumi.26@gmail.com (S.H.); esuzuki@o.cc.titech.ac.jp (E.-i.S.); fujii.s.ap@m.titech.ac.jp (S.F.); yuji-w@apc.titech.ac.jp (Y.W.); 2 Department of Marine Resource Science, Faculty of Agriculture and Marine Science, Kochi University, Monobe-otsu 200, Nankoku 783-8502, Japan; chuji@kochi-u.ac.jp; 3 Research Institute for Sustainable Humanosphere, Kyoto University, Gokasho, Uji 611-0011, Japan; mitani@rish.kyoto-u.ac.jp; 4 Department of Information and Communication Systems Engineering, Okinawa National College of Technology, 905 Henoko, Nago-shi, Okinawa 905-2192, Japan

**Keywords:** polyoxometalate, dielectric spectroscopy, radio frequency, proton relay

## Abstract

Electromagnetic waves, such as microwaves, have been used to enhance various chemical reactions over polyoxometalates. The dielectric properties of catalysts are among the relevant parameters facilitating catalytic reactions under electromagnetic radiation. This study describes the dielectric properties of polyoxometalate catalysts in aqueous and organic solutions to understand the mechanism of interactions between polyoxometalates and electromagnetic waves. Specific loss factors of polyoxometalates were observed at lower frequencies (<1 GHz) by the ionic conduction of the polyoxometalate solution. The evolution of ionic conduction depended strongly on cations rather than anions. Proton-type polyoxometalates exhibited significantly higher loss factors than other cations did. The activation energy for ionic conduction in protonated silicotungstic acid (H_4_SiW_12_O_40_) was significantly low in water (7.6–14.1 kJ/mol); therefore, the high loss factor of protonated polyoxometalates in water was attributed to the proton relay mechanism (i.e., Grotthuss mechanism). The results suggested that the proton relay mechanism at the radio-frequency band is critical for generating selective interactions of polyoxometalates with applied electromagnetic fields.

## 1. Introduction

Polyoxometalates (POMs) exhibit unique catalysis for many oxidation and reduction reactions, alkene polymerization, and acid and base catalysis reactions [1,2]. POMs comprise oxo-anions of heteroatoms (Si, P, S, Ge, As, Se, B, Al, and Ga) and addenda atoms (W, Mo, V, Nb, and Ta). The general chemical formula for Keggin-type POMs is given by [XM_12_O_40_]^n−^ (X = heteroatoms; M = addenda atoms). Because of the high stability of POM anions, POMs exhibit very low acid dissociation constants; thus, POMs are used as super acid catalysts [1]. At the same time, the multi-electron-transfer redox property of POMs under relatively mild conditions enables selective oxidative catalysis, such as the high-yield syntheses of epoxides and aldehydes [1,2]. The unique catalytic properties of POMs have been applied for many biorefinery processes, such as hydrolysis of crystalline cellulose [3,4], oxidative delignification [5], and the oxidation of biomass for the production of syngas [6], formic acid [7], and hydrogen [8].

We have recently reported that microwave (MW) irradiation enhances the POM-catalyzed hydrolysis of biomass, such as crystalline cellulose [9], seaweeds [10], and cellobiose [11]. Dielectric heating by electromagnetic waves enables the rapid and selective heating of materials by inducing direct interactions with the irradiated materials. MWs have been reported to enhance various chemical processes including organic synthesis [12], inorganic synthesis [13], material processing [14], metal sintering [15], food processing [16], and biorefinery processes [17]. MW irradiation is also effective for POM-catalyzed biomass conversion reactions [18,19] and the synthesis of POMs and POM-related materials [20,21,22,23,24]. The understanding of the mechanism of interaction between POM and electromagnetic waves is, therefore, important for their effective use in enhancing POM-related reactions.

The dielectric heating of materials occurs by various loss mechanisms, including ionic conduction (σ), dielectric loss (ε″), and magnetic loss (μ″) under an applied electromagnetic field. The dielectric heating phenomenon can be expressed by the following equation:

(1) $$P=\frac{1}{2}\sigma {\left|E\right|}^{2}+\pi f\varepsilon_{0}{\varepsilon_{r}}^{\prime \prime }{\left|E\right|}^{2}+\pi f\mu_{0}{\mu_{r}}^{\prime \prime }{\left|H\right|}^{2},$$

where *P*, *E*, *H*, *ε*_0_, and *μ*_0_ are the absorbed power by volume, electric field intensity, magnetic field intensity, permittivity of free space, and permeability of free space, respectively. The dielectric properties and intensities of the electric and magnetic fields are the key parameters to manipulate to obtain the high-efficiency energy propagation of electromagnetic waves. In addition, dielectric properties are strongly related to the molecular dynamics of dipoles and charges (ions and electrons) under oscillating electromagnetic fields [24,25,26,27]. Therefore, the dielectric properties of a material provide information regarding the molecular dynamics of dipoles and charges under electromagnetic fields, as well as the degree of the electromagnetic wave susceptibility of the material.

In this study, the dielectric properties of POMs under applied electromagnetic radiation from the radio-frequency (RF) band to MWs were analyzed to understand the absorption mechanisms of electromagnetic waves by various POMs with different chemical compositions. Dielectric spectroscopy was conducted by the coaxial probe method to characterize the complex dielectric constants of the POMs in different solvents such as water, dimethylsulfoxide (DMSO), and 2-propanol. The dielectric loss mechanism of the POMs was further investigated by applying the Cole–Cole model to reveal the key parameters involved in the dielectric loss mechanisms of POMs in solution.

## 2. Materials and Methods

### 2.1. Materials

Silicotungstic acid (H_4_SiW_12_O_40_·28H_2_O), phosphotungstic acid (H_3_PW_12_O_40_·*n*H_2_O), and phosphomolybdic acid (H_3_PMo_12_O_40_·*n*H_2_O) were purchased from FUJIFILM Wako Pure Chemical Corporation. H_3_AsW_12_O_40_·*n*H_2_O, H_4_S_2_W_18_O_62_·*n*H_2_O, H_4_PVW_11_O_40_·*n*H_2_O, K_4_PVW_11_O_40_·*n*H_2_O, and (NH_4_)_4_PVW_11_O_40_·*n*H_2_O were prepared with the method reported previously [28]. The water contents in the POMs were determined by thermogravimetric analysis (TGA, Shimadzu Co., Kyoto, Japan; Table 1). The POMs were used without further purification.

### 2.2. Dielectric Relaxation Spectroscopy

Dielectric relaxation spectra were obtained by the coaxial probe method using a Keysight 5242A Network Analyzer and a Keysight high-temperature probe (100 MHz–20 GHz) or a Rhode & Schwartz ZND network analyzer equipped with a KEYCOM Co. (Tokyo, Japan) probe-type (open mode) dielectric constant measurement kit (200 MHz–8.5 GHz). The POMs were dissolved in pure water, DMSO, or aqueous 2-propanol (0–100 *v*/*v* %) solutions of 1–20 mM. Dielectric relaxation spectra were obtained between room temperature (25 °C) and 80 °C by changing the temperature using a bath of aluminum beads with stirring to obtain solutions with homogeneous temperatures. The temperature during the dielectric measurement was monitored by a fiber-optic thermometer (FL-2000, Anritsu Meter Co., Ltd., Tokyo, Japan) under stirring to ensure temperature homogeneity of the POM solutions.

## 3. Results and Discussion

### 3.1. Dielectric Properties of POMs in Water, 2-Propanol, and DMSO

**Figure 1:**
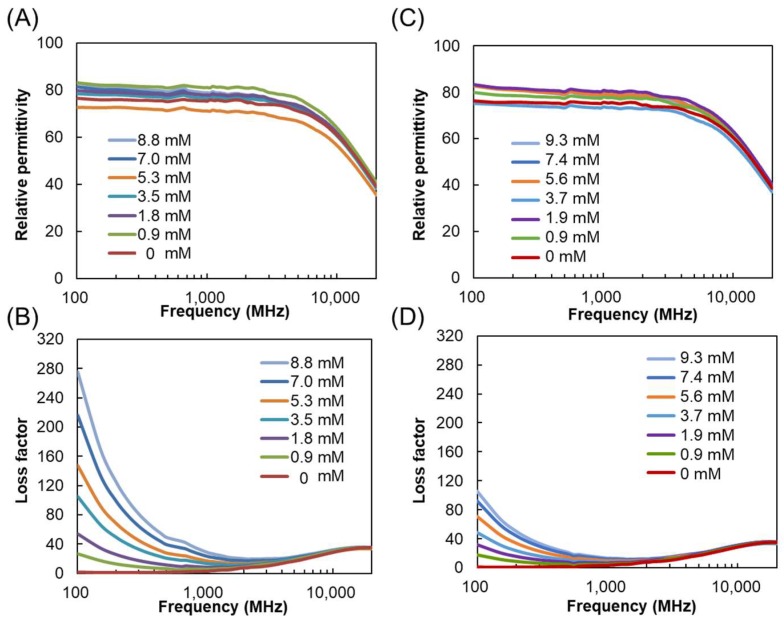
(**A**) Relative permittivity and (**B**) loss factor of H_4_SiW_12_O_40_ in water (0–8.8 mM); (**C**) relative permittivity and (**D**) loss factor of H_3_PW_12_O_40_ in water (0–9.3 mM). Measurement temperature: 26 ± 1 °C.

The complex dielectric constants of the POM solutions were measured by the coaxial method using open probes and a vector network analyzer. The real part and imaginary part of the complex dielectric constant represent the relative permittivity and loss factor, including dielectric loss (ε″) and ionic conduction (σ), respectively. Figure 1A,B show the dielectric spectra of H_4_SiW_12_O_40_ in water (0–8.8 mM) at frequencies of 100 MHz–20 GHz. The loss factors of H_4_SiW_12_O_40_ are strongly affected by the acid concentration. Aqueous H_4_SiW_12_O_40_ solutions show particularly elevated losses at frequencies of <2000 MHz, which becomes more prominent in the RF band (<300 MHz) rather than under MWs. The loss mechanism at this frequency range can be attributed to ionic conduction by electrolytes [26]. In contrast, the dielectric dispersion at higher frequencies, peaking at 18–20 GHz, does not significantly change with concentration. The peak is assigned to the cooperative relaxation of long-range hydrogen-bond-mediated dipole–dipole interactions of free water [29]. The dielectric spectra of H_3_PW_12_O_40_ show similar behavior to those of H_4_SiW_12_O_40_, exhibiting enhanced loss factors at lower frequencies; however, the magnitudes of the loss factors are smaller than those of H_4_SiW_12_O_40_ (Figure 1C,D).

**Figure 2:**
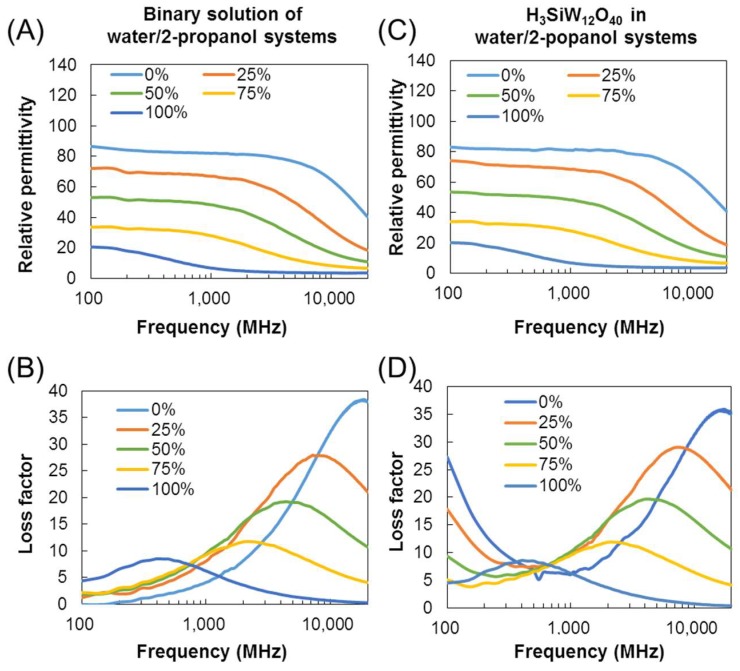
Dielectric properties of POMs in mixed solutions of water and 2-propanol (2-propanol concentrations = 0–100 *v*/*v* %). (**A**) Relative permittivity and (**B**) loss factor of binary system of water and 2-propanol; (**C**) relative permittivity and (**D**) loss factor of H_4_SiW_12_O_40_ in mixed solutions of 2-propanol and water (1 mM). Measurement temperature: 26 ± 1 °C.

Figure 2 shows the dielectric spectra of binary systems of 2-propanol and water with and without H_4_SiW_12_O_40_. The ratios of 2-propanol to water were varied to investigate the effect of the relative permittivity of the solvent on the dielectric properties of the POM solution. The relative permittivity is decreased from 86.6 to 20.6 with increased 2-propanol concentration (Figure 2A) [30,31]. Along with the decrease in relative permittivity, the dielectric loss peak is shifted from 18–20 GHz (free water) to 300–400 MHz (2-propanol) with a reduction in peak intensity (Figure 2B). The loss factor by ionic conduction is gradually weakened by increased 2-propanol concentration, and is buried by the dielectric loss of 2-propanol (Figure 2D). The decrease in ionic conduction indicates that the dissociation of H_4_SiW_12_O_40_ should be prohibited by the addition of 2-propanol. The dissociation of an acid depends on the dielectric constants and proton affinities of the solvents granted by their molecular structures [32]. Smaller dielectric constants provide larger Coulomb potentials, thus prohibiting ionic dissociation. Because 2-propanol has a lower relative permittivity than water, the dissociation constants of acids become higher than those in water.

**Figure 3:**
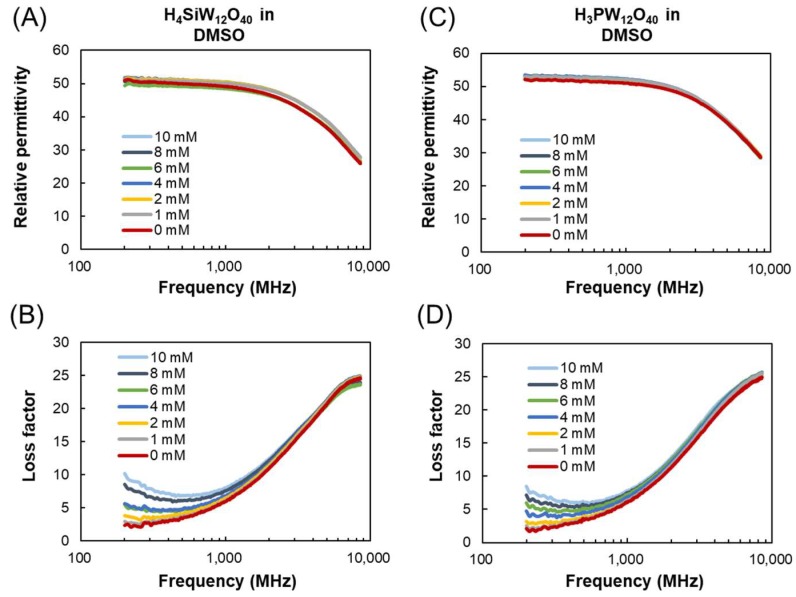
Dielectric properties of POMs in DMSO. (**A**) Relative permittivity and (**B**) loss factor of H_4_SiW_12_O_40_ in DMSO (0–10 mM); (**C**) relative permittivity and (**D**) loss factor of H_3_PW_12_O_40_ in DMSO (0–10 mM). Measurement temperature: 26 ± 1 °C.

Figure 3 shows dielectric spectra of H_4_SiW_12_O_40_ and H_3_PW_12_O_40_ in DMSO. DMSO was found to exhibit a specific solvent effect on the oxidation of alcohol via PV*_x_*Mo_(12−*x*)_O_40_^−(3+*x*)^ (*x* = 0, 2) because DMSO works as an oxygen donor during the reaction [33]. Similar to the case in the aqueous POM solutions, the relative permittivity does not significantly change with increasing POM concentration. The peak for the dielectric loss of DMSO at ~8 GHz is not obviously affected by the different concentrations of POMs from 0 to 10 mM. In contrast, the dielectric losses at lower frequencies are increased with increased concentrations of H_4_SiW_12_O_40_ and H_3_PW_12_O_40_ in DMSO by ionic conduction. H_4_SiW_12_O_40_ has a slightly higher loss factor than H_3_PW_12_O_40_; however, the ionic conduction by POMs in DMSO is much smaller than that in water because of the lower conductivities of the electrolytes in DMSO. The dissociation constant of POMs in DMSO should be between those in water and 2-propanol, because the relative permittivity of DMSO (46.9) is between those of water (86.6) and 2-propanol (20.6).

The above results indicate that the loss factors of POMs in a solution are selectively increased at lower frequencies corresponding to the RF band. The dissociation of POMs is required to initiate clear ionic conduction in the dielectric spectra. A relative permittivity of >50 (such as those of 50% aqueous 2-propanol solution and DMSO) is essential for obvious ionic conduction by the dissociation of POM in the solvents. For the molybdenum-based Keggin POM of H_3_PMo_12_O_40_, the dependence of dielectric properties on solvents were in very good accordance with those of H_4_SiW_12_O_40_ and H_3_PW_12_O_40_ (Figures S1 and S2). Therefore, solvents with relatively high permittivities, such as water, are suitable for improving the specific dielectric losses of POMs.

### 3.2. Mechanism of Interaction of POMs and Electromagnetic Waves in Water

The mechanism of interaction between the POMs and electromagnetic field was further studied by applying the dielectric relaxation model to the dielectric parameters, allowing quantitative evaluation of the POM conductivity in solvents and of the solvent relaxation process. The real and imaginary parts of the complex dielectric constants were further analyzed by a dielectric relaxation model. A general dielectric relaxation model is given by the Debye model (2):

(2) $$\varepsilon_{r}^{*}=\varepsilon_{\infty }+\frac{\varepsilon_{s}-\varepsilon_{\infty }}{1+j\omega \tau },$$

where ε*, *ε*_s_, *ε*^∞^, *j*, *ω*, and *τ* are the complex permittivity, the permittivity at a static frequency, the permittivity at infinite frequency, the imaginary unit, angular frequency, and relaxation time, respectively. The Debye model can be applied to single components. The Cole–Cole model (3) is more suitable for mixed solutions of more than two components by introducing *β* (0 < *β* ≤ 1) as a distribution parameter:

(3) $$\varepsilon_{r}^{*}=\varepsilon_{\infty }+\frac{\varepsilon_{s}-\varepsilon_{\infty }}{1+{\left(j\omega \tau \right)}^{\beta }}.$$

For a solution containing an electrolyte, a term for the contribution of ionic conduction can be applied to the Cole–Cole model as follows (4):

(4) $$\varepsilon_{r}^{*}=\varepsilon_{\infty }+\frac{\varepsilon_{s}-\varepsilon_{\infty }}{1+{\left(j\omega \tau \right)}^{\beta }}-\frac{j\sigma }{\omega \varepsilon_{0}},$$

where *ε*_0_ and *σ* are the permittivity of free space and conductivity of the electrolyte, respectively. 

**Figure 4:**
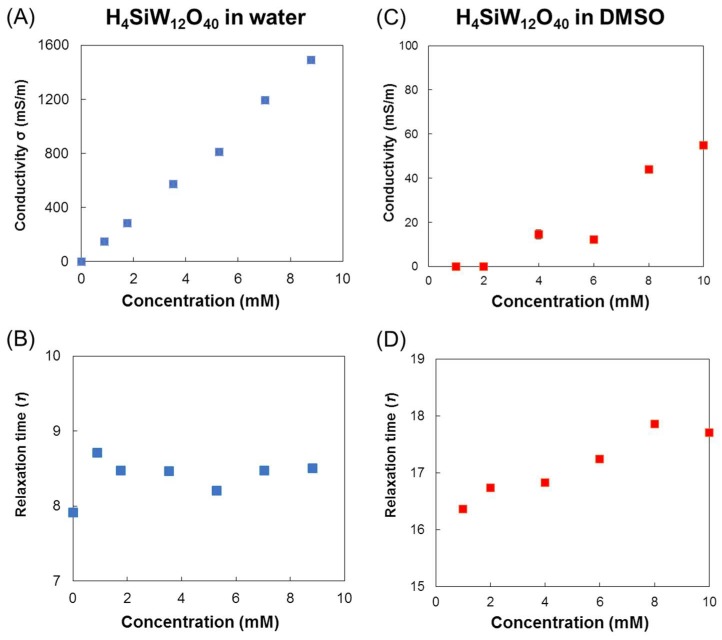
Dependence of conductivities (H_4_SiW_12_O_40_) and relaxation times (water and DMSO) on the concentration of H_4_SiW_12_O_40_ (0–10 mM). (**A**) Conductivity of H_4_SiW_12_O_40_ in water as a function of its concentration; (**B**) conductivity of H_4_SiW_12_O_40_ in dimethylsulfoxide as a function of its concentration; (**C**) relaxation time of water as a function of H_4_SiW_12_O_40_ concentration; and (**D**) relaxation time of DMSO as a function of H_4_SiW_12_O_40_ concentration. Measurement temperature: 26 ± 1 °C.

The conductivity and the dielectric relaxation time were calculated by fitting the Cole–Cole model considering the conductivity, as shown in Equation (4). The conductivities of H_4_SiW_12_O_40_ in water and DMSO are indicated in Figure 4A,C. The increased concentration of H_4_SiW_12_O_40_ in water linearly increases the conductivity. In contrast, a concentration of >5 mM is required for H_4_SiW_12_O_40_ to exhibit conductivity in DMSO. The conductivity of H_4_SiW_12_O_40_ in water is much higher than that in DMSO. The relaxation times for water and DMSO are shown in Figure 4B,D. In aqueous solutions, the relaxation time of water remains almost constant between 8 and 8.5 ps. However, the relaxation time of DMSO is linearly increased from 16 to 18 ps with increased H_4_SiW_12_O_40_ concentration. Therefore, the restriction of the molecular motion of DMSO is gradually strengthened by the addition of H_4_SiW_12_O_40_. This indicates strong interactions between DMSO and H_4_SiW_12_O_40_. H_3_PW_12_O_40_ and H_3_PMo_12_O_40_ also showed very similar tendencies in DMSO and water (Figure S3).

**Figure 5:**
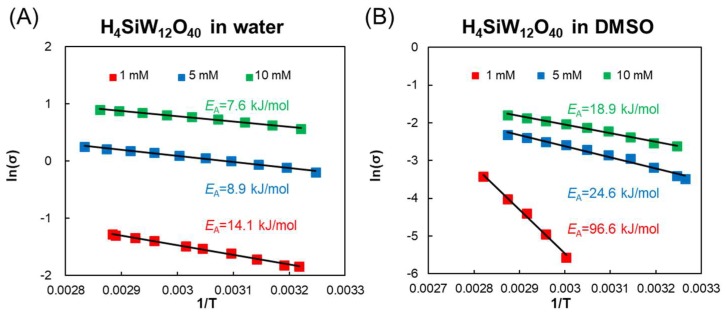
Arrhenius plots of conductivities of POMs. (**A**) H_4_SiW_12_O_40_ in water (1, 5, and 10 mM); (**B**) H_4_SiW_12_O_40_ in DMSO (1, 5, and 10 mM).

The temperature dependence of conductivities is shown in Figure 5. The elevated temperature increases the conductivity of H_4_SiW_12_O_40_ in both water and DMSO. The temperature-dependent conductivities are further fitted to the Arrhenius Equation (5) to obtain the activation energy (*E*_a_) of the conduction process:

(5) $$\ln\left(\sigma \right)=\ln\left(A\right)-\frac{E_{a}}{RT},$$

where *A*, *R*, and *T* are the frequency factor, ideal gas constant, and absolute temperature, respectively.

The activation energy of conductivity in water is estimated at 14.1 kJ/mol, while that in DMSO (1 mM) is ~96.6 kJ/mol (Figure 5). The higher activation energy of H_4_SiW_12_O_40_ in DMSO may be due to the constraint of ionic conduction by the strong interaction of DMSO and H_4_SiW_12_O_40_. By increasing the concentration of H_4_SiW_12_O_40_ to 5–10 mM, the activation energy is decreased to 8.9 kJ/mol (5 mM)–7.6 kJ/mol (10 mM) in water and to 24.6 kJ/mol (5 mM)–18.9 kJ/mol (10 mM) for DMSO. POMs are well-known highly proton-conductive materials, often used in proton-exchange membrane fuel cells [34]. Horky et al. previously reported the infinite dilution conductivity of cations (Λ° H_3_O^+^) and POM anions (Λ° anion) for H_4_SiW_12_O_40_ and H_3_PW_12_O_40_ in water [35]. The low conductivity of POM anions indicated that their conduction entirely relies on hydrodynamic transport (the vehicle mechanism). However, the large mobility of protons (H_3_O^+^) arises from the proton relay mechanism (or Grotthuss mechanism), which involves fast proton transport by the consecutive formation and destruction of H_3_O^+^. Wang et al. reported that the activation energy for conductivity in H_3_PW_12_O_40_ (0.6–6.7 mM) and H_4_SiW_12_O_40_ (0.13–3 mM) in dimethyl formamide (DMF) is ~7.9–8.3 kJ/mol [33]. The activation energy of <15 kJ/mol indicates the proton relay mechanism, whereas that of >20 kJ/mol is due to the vehicle mechanism. Therefore, dielectric loss of H_4_SiW_12_O_40_ in water under RF-band electromagnetic waves can be attributed to fast proton conduction. In DMSO, the conduction process is much lower than that in water, indicating the importance of water in enabling high dielectric loss by the proton relay mechanism.

**Figure 6:**
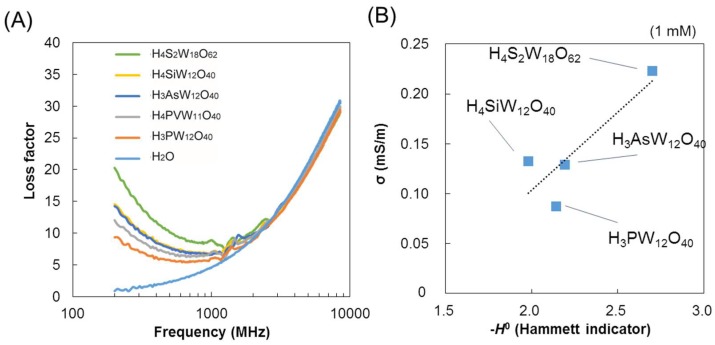
(**A**) The dielectric spectra of five types of POM in water (1 mM); (**B**) correlation of conductivity of POMs and acidity (Hammett indicator) [35].

The dielectric properties of several types of POM with different compositions and structures are analyzed in water (1 mM) (Figure 6A). The degree of ionic conduction varies depending on the type of POM. H_4_S_2_W_18_O_62_, a Wells–Dawson-type POM, shows the highest ionic conduction (0.223 S/m), followed by H_4_SiW_12_O_40_ (0.133 S/m) and H_3_AsW_12_O_40_ (0.129 S/m). The conductivities of the POMs are further plotted over their acidities, as described by the Hammett indicator (Figure 6B) [28]. The acidities of the POMs show good accordance with their conductivities because of the higher concentrations of protons in the systems. Therefore, the degree of acid dissociation is another important factor to obtain better ionic conductivity by POMs.

**Figure 7:**
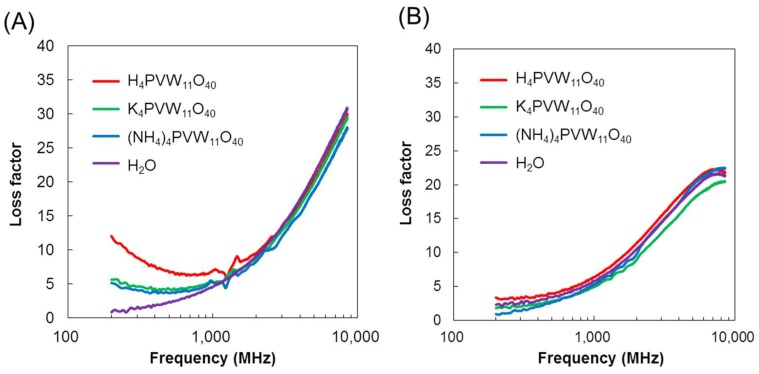
Cation-dependent dielectric relaxation spectra of [PVW_11_O_40_]^4−^ POM anion (1 mM) (**A**) in water and (**B**) in DMSO.

To investigate the contribution of cations to the loss factors of POMs, the dielectric spectra of the [PVW_11_O_40_]^4−^ anion with different cations (H^+^-, K^+^-, and (NH_4_)^+^-forms) are measured in aqueous solutions (Figure 7). The protonated [PVW_11_O_40_]^4−^ exhibit the highest ionic conductivity, reaching 0.141 S/m, while those for the K^+^- and (NH_4_)^+^-forms are only 0.040 S/m and 0.055 S/m, respectively. Therefore, the large conductivity of protons significantly affects the loss factors of POM solutions. In DMSO at 1 mM, however, no proton-dependent enhancement was observed. These results coincide with the large activation energy required for ionic conduction at low POM concentrations in DMSO, compared to the very small activation energies required in water (Figure 5). As a summary, protonated POMs exhibit specific loss factors at lower frequencies in aqueous media than in non-aqueous ones because of the proton relay mechanism.

Interestingly, we have previously observed similar proton-enhanced ionic conductivity in an aqueous system of natural polysaccharides obtained from seaweeds [36,37]. The dielectric properties of acidic polysaccharides, such as *κ*- and λ-carrageenan (sulfated galactan), are significantly affected by the conductivities of their cations. When the cation of carrageenan was exchanged from the K^+^-form (as-prepared) to the proton-form by a strong cation-exchange resin, a significant enhancement of ionic conductivity was observed in the RF band (<300 MHz). Therefore, the proton-enhanced ionic conductivity may be a general phenomenon in many materials. Ionic conductivity is among the key parameters needed to obtain efficient dielectric heating, as described in Equation (1). Because the power of dielectric heating strongly depends on the conductivity, ionic conductivity has been used to improve the MW susceptibility of reaction systems [38,39,40]. The presented results indicate that stronger and more selective interactions can be obtained between protonated POMs and electromagnetic waves by applying RF fields rather than MWs.

## 4. Conclusions

Electromagnetic waves have been used to activate various chemical reactions catalyzed by POMs. The dielectric properties of irradiated materials are important factors in obtaining good energy propagation during reactions under electromagnetic fields. The dielectric properties of POMs were, therefore, characterized by dielectric spectroscopy under fields at frequencies between the RF band and MWs in this study. Selective loss factor increases of POMs in water were observed at frequencies below <1 GHz; these became more prominent in the RF band (<300 MHz). This process was due to the ionic conductivity of the POMs and strongly depended on their concentration. Dielectric constants of >50 were required to initiate POM dissociation and thus ionic conductivity. The very low activation energy for ionic conductivity by POMs indicated that the proton relay mechanism is involved in the dielectric loss of POMs. The contribution of the proton relay mechanism was further confirmed by varying the acid strength and cations of POMs. Namely, POMs with greater acidity exhibited more intense ionic conduction. In addition, the protonated POM exhibited larger ionic conductivity than the K^+^-form and NH_4_^+^-form. Because a similar proton relay mechanism was previously observed for protonated natural polysaccharides, this phenomenon may be a general mechanism during the dielectric heating of many electrolyte materials.

## Figures and Tables

**Table 1:** Numbers of hydration in polyoxometalates used in this study.

POM	Hydration Number
H_4_SiW_12_O_40_	28
H_3_PW_12_O_40_	25
H_3_PMo_12_O_40_	28
H_3_AsW_12_O_40_	10
H_4_PVW_11_O_40_	10
H_4_S_2_W_18_O_62_	27
(NH_4_)_4_PVW_11_O_40_	9
K_4_PVW_11_O_40_	4

## Supplementary Materials

The following are available online at http://www.mdpi.com/1996-1944/11/7/1202/s1, Figure S1: Dielectric properties of POMs in mixed solutions of water and 2-propanol (2 propanol concentration; 0–100 *v*/*v* %). (A) Relative permittivity and (B) loss factor of H_3_PMo_12_O_40_ in mixed solutions of 2-propanol and water (H_3_PMo_12_O_40_; 1 mM), Figure S2: (A) Relative permittivity and (B) loss factor of H_3_PMo_12_O_40_ in DMSO (0–10 mM), Figure S3: Dependencies of conductivities (POM) and relaxation times (solvent) on POM concentrations and temperature. (A) Conductivity of H_3_PW_12_O_40_ in water, (B) relaxation time of H_3_PW_12_O_40_ in water, (C) conductivity of H_3_PW_12_O_40_ in DMSO, (D) conductivity of H_3_PW_12_O_40_ in DMSO, (E) relaxation time of water as a function of concentration of H_3_PMo_12_O_40_, and (F) relaxation time of DMSO as a function of concentration of H_3_PMo_12_O_40_ in DMSO.
