# Supplementary material for: Proton-Enhanced Dielectric Properties of Polyoxometalates in Water under Radio-Frequency Electromagnetic Waves
Source: Materials (Basel). 2018 Jul 13;11(7):1202. doi: 10.3390/ma11071202 (PMC6073116; doi:10.3390/ma11071202)
Supplement: Supplementary file 1 [file materials-11-01202-s001.pdf]

Supplementary Materials

Proton-Enhanced Dielectric Properties of Polyoxometalates in Water under Radio-Frequency Electromagnetic Waves

Shuntaro Tsubaki [1,*], Shogo Hayakawa [1], Tadaharu Ueda [2], Tomohiko Mitani [3], Ei-ichi Suzuki [1], Satoshi Fujii [1,4] and Yuji Wada [1]

[1] School of Materials and Chemical Technology Tokyo Institute of Technology, Ookayama 2-12-1 E4-3, Meguro, Tokyo 152-8550, Japan; tetuya.utumi.26@gmail.com (S.H.); esuzuki@o.cc.titech.ac.jp (E.-i.S.); fujii.s.ap@m.titech.ac.jp (S.F.); yuji-w@apc.titech.ac.jp (Y.W.)

[2] Department of Marine Resource Science, Faculty of Agriculture and Marine Science, Kochi University, Monobe-otsu 200, Nankoku 783-8502, Japan; chuji@kochi-u.ac.jp

[3] Research Institute for Sustainable Humanosphere, Kyoto University, Gokasho, Uji 611-0011, Japan; mitani@rish.kyoto-u.ac.jp

[4] Department of Information and Communication Systems Engineering, Okinawa National College of Technology, 905 Henoko, Nago-shi, Okinawa 905-2192, Japan

* Correspondence: tsubaki.s.aa@m.titech.ac.jp, shuntaro.tsubaki@gmail.com ; Tel.: +81-3-5734-3735

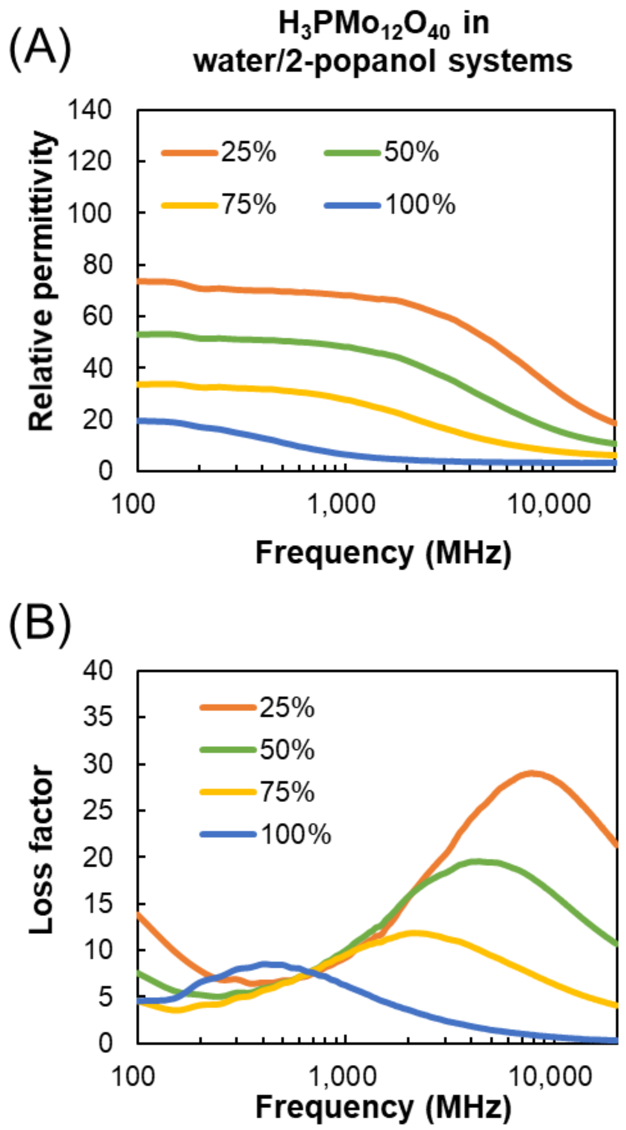

Figure S1. Dielectric properties of POMs in mixed solutions of water and 2-propanol (2 propanol concentration; 0–100 *v*/*v* %). (A) Relative permittivity and (B) loss factor of $H_3PMo_{12}O_{40}$ in mixed solutions of 2-propanol and water ($H_3PMo_{12}O_{40}$; 1 mM).

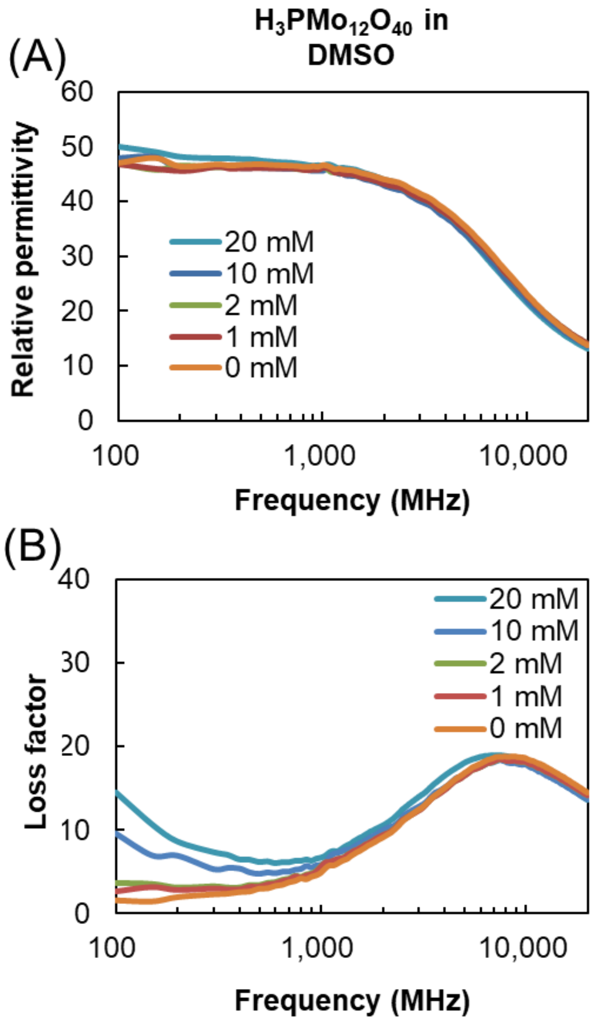

Figure S2. (A) Relative permittivity and (B) loss factor of $H_3PMo_{12}O_{40}$ in DMSO (0–10 mM).

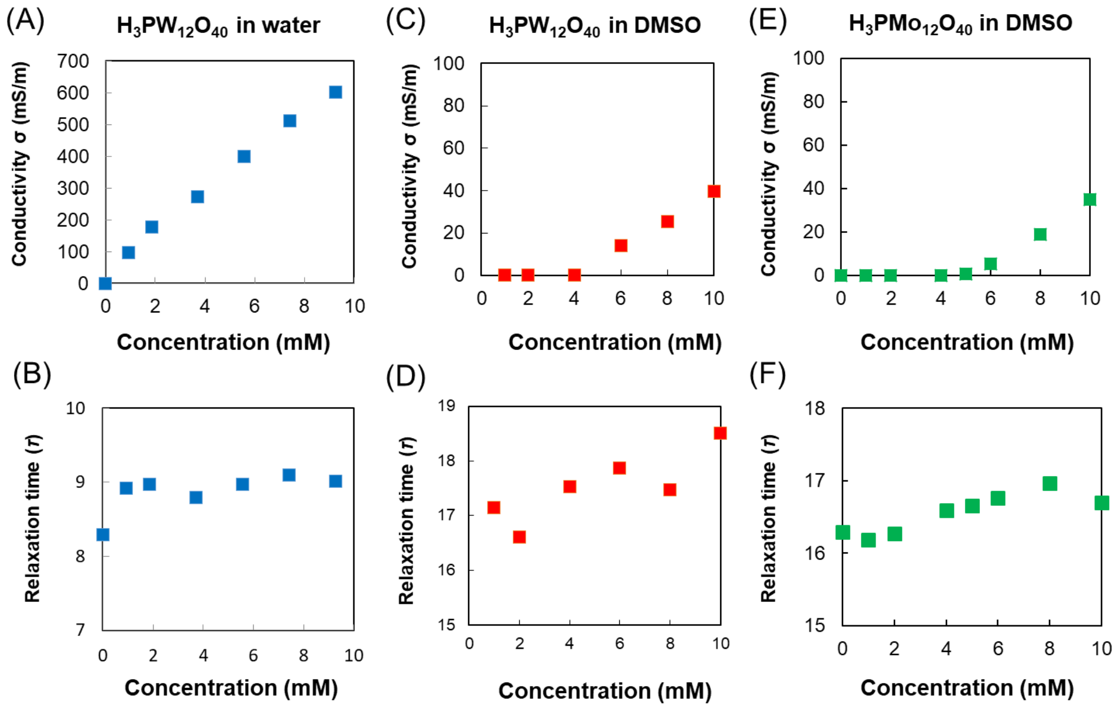

Figure S3. Dependencies of conductivities (POM) and relaxation times (solvent) on POM concentrations and temperature. (A) Conductivity of $H_3PW_{12}O_{40}$ in water, (B) relaxcation time of $H_3PW_{12}O_{40}$ in water, (C) Conductivity of $H_3PW_{12}O_{40}$ in DMSO, (D) conductivity of $H_3PW_{12}O_{40}$ in DMSO, (E) relaxation time of water as a function of concentration of $H_3PMo_{12}O_{40}$, and (F) relaxation time of DMSO as a function of concentration of $H_3PMo_{12}O_{40}$ in DMSO.
